# Kinetic Memory Based on the Enzyme-Limited Competition

**DOI:** 10.1371/journal.pcbi.1003784

**Published:** 2014-08-14

**Authors:** Tetsuhiro S. Hatakeyama, Kunihiko Kaneko

**Affiliations:** Department of Basic Science, Graduate School of Arts and Sciences, The University of Tokyo, Komaba, Meguro-ku, Tokyo, Japan; Chapman University, United States of America

## Abstract

Cellular memory, which allows cells to retain information from their environment, is important for a variety of cellular functions, such as adaptation to external stimuli, cell differentiation, and synaptic plasticity. Although posttranslational modifications have received much attention as a source of cellular memory, the mechanisms directing such alterations have not been fully uncovered. It may be possible to embed memory in multiple stable states in dynamical systems governing modifications. However, several experiments on modifications of proteins suggest long-term relaxation depending on experienced external conditions, without explicit switches over multi-stable states. As an alternative to a multistability memory scheme, we propose “kinetic memory” for epigenetic cellular memory, in which memory is stored as a slow-relaxation process far from a stable fixed state. Information from previous environmental exposure is retained as the long-term maintenance of a cellular state, rather than switches over fixed states. To demonstrate this kinetic memory, we study several models in which multimeric proteins undergo catalytic modifications (e.g., phosphorylation and methylation), and find that a slow relaxation process of the modification state, logarithmic in time, appears when the concentration of a catalyst (enzyme) involved in the modification reactions is lower than that of the substrates. Sharp transitions from a normal fast-relaxation phase into this slow-relaxation phase are revealed, and explained by enzyme-limited competition among modification reactions. The slow-relaxation process is confirmed by simulations of several models of catalytic reactions of protein modifications, and it enables the memorization of external stimuli, as its time course depends crucially on the history of the stimuli. This kinetic memory provides novel insight into a broad class of cellular memory and functions. In particular, applications for long-term potentiation are discussed, including dynamic modifications of calcium-calmodulin kinase II and cAMP-response element-binding protein essential for synaptic plasticity.

## Author Summary

Cellular memory exists in a wide range of organisms from prokaryotes to eukaryotes and can persist, in some cases, for days at a time. Mounting evidence supports the notion that cells indeed can retain information associated with previous environmental exposures via posttranslational modifications. Molecules with multiple modification sites, such as those regulated by changes in methylation or phosphorylation, are expected to play an important role in this process. However, how such epigenetic cellular memory is preserved is not yet fully understood, and theoretical models must be developed. Here we demonstrate that long-term maintenance of the modification state occurs as a result of enzyme-limited competition in a wide class of multimeric protein systems consisting of modification reactions that share enzymes. This maintenance is explained by very slow relaxation resulting from the limited amount of enzymes available. Therefore, memory is easily affected by continuous changes in external stimuli. The proposed mechanism for kinetic memory does not require any fine-tuning in system parameters and is applied to a broad class of cellular memory including long-term potentiation of synapses.

## Introduction

The importance of cellular memory, in which information from experienced environmental exposures is preserved within cellular states, has received a great deal of attention in recent years. The capability of cells to translate environmental exposures into cellular memory has been reported in various organisms, ranging from bacteria to unicellular protozoa and multicellular vertebrates [1]–[5]. Such examples of cellular memory are thought to result from stored epigenetic changes that are not restricted to histone modifications but rather include long-term modifications (e.g., phosphorylation, methylation, and acetylation) of proteins and DNAs that regulate gene expression and thereby affect cellular states [6]–[9]. Generally, cellular epigenetic memory is regarded to occur more slowly than elementally biochemical reactions without affecting the genome sequence, and is considered to be important for various cellular functions, such as adaptation to external stimuli, cell differentiation, and synaptic plasticity [1], [4], [5], [9], [10].

One of the most prominent examples of cellular memory is long-term potentiation (LTP) for synaptic plasticity, which is defined by an increase in the synaptic strength over a long time span. Persistent phosphorylation of 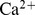/calmodulin-dependent protein kinase II (CaMKII) is known to be particularly important in early LTP [6], [7]. CaMKII phosphorylation is elevated by transient increases in the concentration of 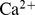 and sustained even after decreases in the concentration of 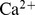. Similarly, in late LTP, the persistent phosphorylation of transcription factors such as cyclic AMP-response element-binding protein (CREB) is important [6], [8]. Another important example of cellular memory is found in the determination of cell fates. For example, when nerve growth factor is administrated to PC12 cells, extracellular-signal-regulated kinase (ERK) is persistently phosphorylated and transmits information to downstream molecules, eventually leading to cell differentiation [9].

Such cellular memory consists of three events: induction, maintenance, and expression. Signaling input can modify the state of the molecule in question, and the modification can be maintained over a time span longer than the time scale of elemental biochemical reactions (e.g., minutes to days). Such modifications can result in changes in expression or in the activation of other molecules. In this study, we focus on the question of how modified states are maintained over a long duration.

One possible avenue for addressing this question is the use of the attractor concept, which includes the “memories-as-attractors” viewpoint whereby dynamical systems governing the modification states have multiple attractors (steady states), in each of which memory is stored as a stable modification state of the substrates [11]–[14] (see Fig. S1A). For example, models for the persistent phosphorylation of CaMKII have been proposed in which the phosphorylation can take on two stable states, such that hysteresis appears against the change in 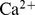 [15]–[17]. In an *in vitro* experiment, however, CaMKII did not show bistability; it only showed ultrasensitivity against the change in 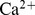 [18]. There have been reports that modification levels are shifted continuously upon stimulation, rather than taking only a few discrete states [18] (see also [19]); this continuity of modification states cannot be explained by the multistability model. Furthermore, the relaxation time: 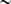 20 minutes after inhibition of CaMKII in ref [20] suggests long-term dynamics without resorting to the bistability discussed therein. In addition, the inclusion of positive feedback processes in gene expression dynamics, which are necessary for the maintenance of multiple states, requires energy, and as a result, memory retention incurs housekeeping costs. In summary, the stability of time-invariant attractors is important in some cases, but in other cases it cannot be explained by the “memories-as-attractors” viewpoint.

Thus, it is important to identify other forms of memory that allow very slow changes of cellular states following transient stimuli. In this respect, we propose “a kinetic memory hypothesis” for epigenetic cellular memory. In this scheme, memory is stored as a slow relaxation process far from an attractor, in which slowness enables long-term maintenance of an embedded state (see Fig. S1B). When cells are stimulated, their cellular states are shifted continuously and are thus kept apart from attractors, and relaxation occurs more slowly than with elemental chemical reactions. Based on this slowness in relaxation, stimulus-induced excited states are memorized over long time spans. This is in strong contrast to the memory-as-attractor scheme, in which new stable states are generated or in which different attractors are selected to store the memory of the stimulus. In kinetic memory, states can be changed continuously depending on the magnitude of the stimulus, and thus epigenetic kinetic memory is more flexible than attractor memory. However, to date, an explicit biochemical model that realizes kinetic memory has not been proposed. In this study, we apply the kinetic memory scheme to model processes underlying cellular memory.

## Results

### Chained modification model

**Figure 1:**
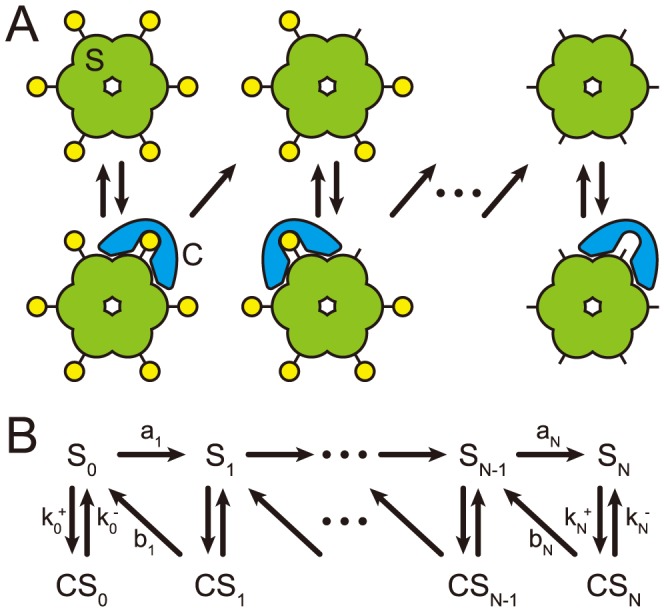
The reaction scheme of the chained modification model. Schematic representation (A) and reaction diagram (B) of our model. A substrate has 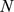 modification sites. Modification reactions for the substrates progress without catalyst at rates 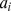 and demodification reactions are facilitated by the catalyst at rates 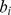.

At first, we introduce a chained modification model showing very slow kinetics (Fig. 1). This model consists of a substrate with 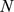 modification sites and a catalyst, where 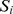 and 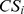 denote the substrates and a substrate-catalyst complex with 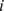-th modified sites, respectively, and 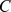 denotes the catalyst. The total abundance of each is defined as conservation quantities given as 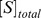 and 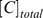, respectively. The use of ‘modification’ here refers to changes such as phosphorylation and methylation. In the first model we study, the catalyst can only facilitate each demodification reaction of the substrate, which is achieved by assuming that enzymes used in modification reactions are always maintained at sufficiently high levels so as not to be rate-limiting. Or, the first-order kinetics of the modification reaction may be considered to be derived from the autophosphorylation reaction (e.g., CaMKII phosphorylation reaction). However, the same results can be obtained, even though both the modification and demodification are catalytic reactions as described below in the kinase-phosphatase model.

The whole reactions in the chained modification model are described below.
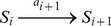


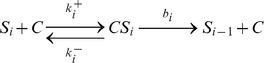
where 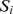 and 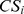 denote the substrates and a substrate-catalyst complex with 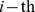 modified sites, respectively, and 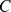 denotes the catalyst.

Here, the formation and dissociation of substrate-catalyst complexes occur at much faster rates than other reactions (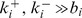) and these reactions are therefore eliminated adiabatically. (We have also confirmed the validity of the approximation numerically.)

By denoting the concentration of free catalyst that does not form a complex as 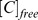 and the total concentration of 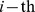 modified substrate, i.e, summation of the concentration of free substrate and substrate-catalyst complex, as 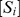, the model can be described as:
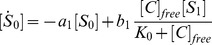


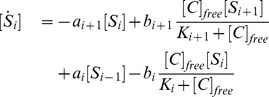


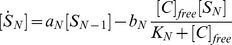
(1)

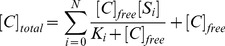
(2)where 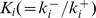 are the dissociation constants between 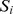 and 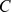. For simplicity, the rate 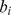 is chosen to be independent of 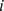, as 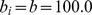.

Generally, the binding energy of complexes depends on the modification sites. For simplicity, we assume that binding energy is reduced linearly per single modification [21], but as long as the binding energy distribution is not narrow (this condition is described below), a deceleration of the relaxation is obtained. The change in affinity is natural, as the modification generally changes the function and shape of the protein [22]. With this simplification, the dissociation constant between 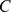 and 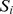 increases exponentially as 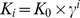, where 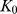 is 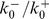. The input is given as changes in the speed of the modification reactions, expressed as 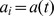 for all 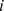 (see Table 1 in Text S1). This is a simplified model for reactions with several modification sites, as discussed in the context of, e.g., phosphorylation and methylation of proteins, whereas several extensions will be discussed later.

### The chained modification model shows slow “glassy” relaxation

To analyze the relaxation process from a highly modified state to a minimally modified state, we set the initial condition as 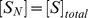 and 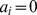 for all 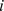. Then, only demodification reactions progress. Under this condition, a modification level (
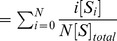
) relaxes from 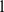 to 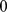 in a single direction. When the concentration of the catalyst is sufficiently high (i.e., in the limit 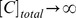), the behavior of this model is same as that of the first-order reactions, such that fast exponential relaxation occurs.

**Figure 2:**
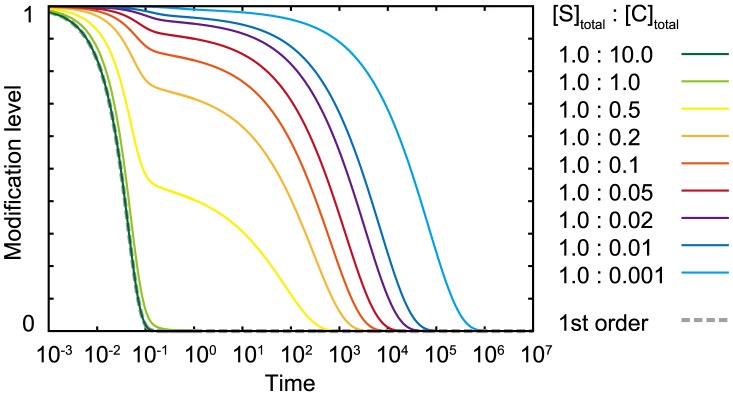
Slow logarithmic relaxation of the chained modification model. The initial condition is set as 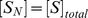 and 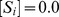 for other 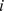, and the relaxation process of the modification level is computed without input (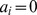). The parameters are given as 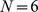, 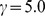, and 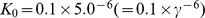. The time courses for the modification level for different values of the catalyst concentration, 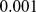, 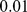, 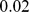, 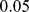, 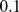, 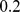, 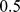, 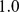, and 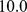, are plotted with different colors, where the concentration of 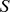 is fixed at 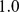. Although exponential relaxation is observed as in first-order reactions (dotted line) when the concentration of the catalyst is sufficiently large, the relaxation is drastically slowed as the concentration of the catalyst becomes lower than that of the substrate.

At low catalyst concentrations, however, the relaxation process is quite different. In this case, as shown in Fig. 2, the modification level relaxes more slowly; it cannot be fitted by the exponential form 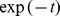 (see Fig. S2A) and is better fitted by the form of 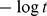 in a certain range (see Fig. S2B), which is termed as the logarithmic relaxation in time. Moreover, when the concentration of the catalyst is within a particular range, the relaxation process shows a plateau. Indeed, such logarithmic relaxation processes and an emergence of a plateau have been shown to exist in glasses [23]. In (statistical) physics, glasses are known to exhibit a very slow relaxation dynamics with a plateau before reaching the final (equilibrium) state, while their long-term relaxation course is fitted by 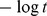 [23], [24]. The origin of such slow relaxation is attributed to kinetic constraint [24], [25]. In this sense, the present relaxation is also referred to “glassy” relaxation.

### The chained modification model shows a transition from slow relaxation to fast relaxation

**Figure 3:**
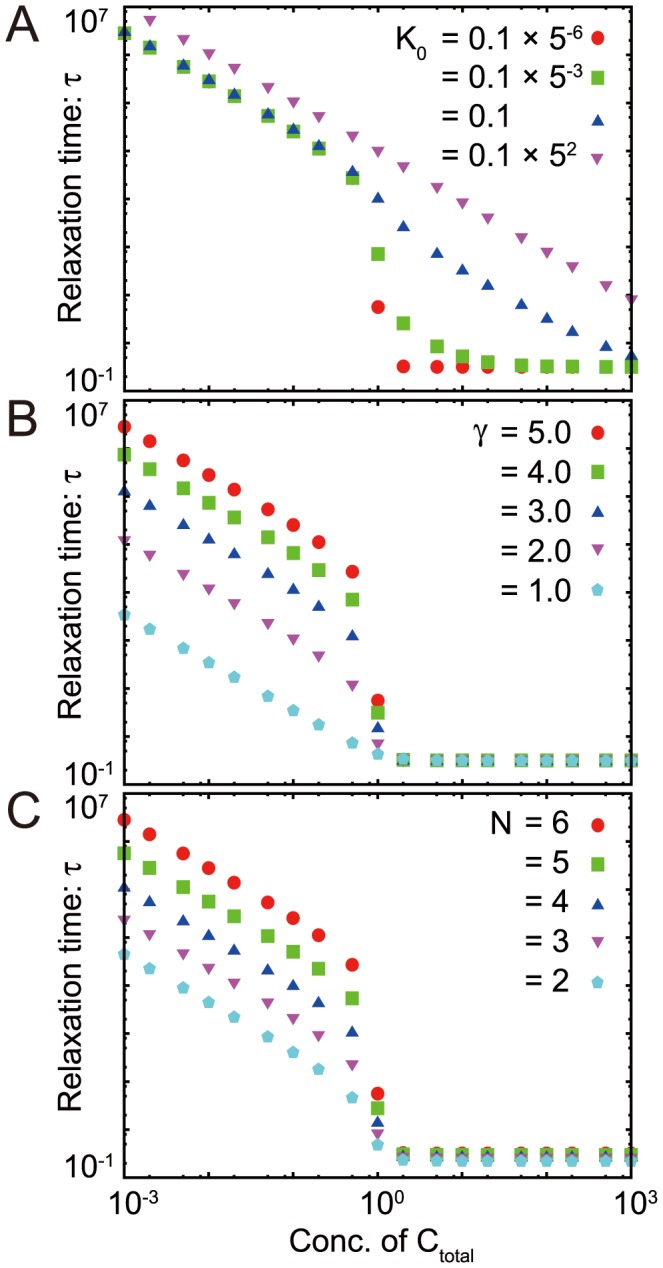
Change in the dependence of the catalyst on the relaxation time 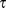 against 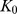, the dissociation constant (A), against 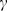, the heterogeneity of the dissociation constant (B), and against 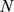, the number of modification sites (C). 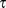
 is plotted against 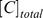; 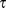 is defined as the time when the summation of all of 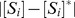 falls below the threshold value(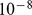) without input, starting from the initial condition (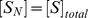). (A) 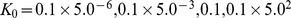, 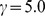, 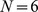. (B) 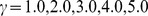, 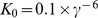, 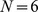. (C) 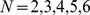, 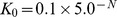, 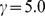.

As shown in Fig. 2, the relaxation process shows a transition from a fast exponential relaxation phase to a slow logarithmic relaxation phase as the catalyst concentration is decreased. To examine this transition quantitatively, we analyzed the dependence of the relaxation time 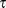 on the concentration of the catalyst. In Fig. 3, the dependence of 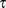 upon the total concentration of catalyst 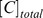 is plotted, and a sudden increase of 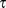 with the decrease of 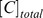 below the concentration of total substrates 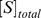 can be observed. Although nonlinear dependence on the catalyst concentration is expected based on Michaelis-Menten kinetics, this sharp increase in 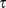 near the critical point results from limitation of the catalyst.

Here, by slightly decreasing the concentration of the catalyst below that of the substrate or by increasing the concentration of the substrate beyond that of the catalyst, the relaxation time suddenly increases by several orders of magnitude. In other words, the relaxation time becomes much longer than the elemental chemical time scale, such that the modification state remains almost at the original level. Hence, the modification state is “memorized” over a long time scale, and storing or erasing memory is achieved by slightly changing the ratio of catalyst to substrate below and beyond the critical value. In summary, our model exhibits a transition from fast exponential relaxation to slow logarithmic relaxation, thereby providing kinetic memory.

### Conditions for kinetic memory

In this section, we discuss the conditions for the existence of a sharp transition to memorized states.

#### (i) The binding affinity should be small – condition for 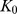


First, we analyzed the dependence of 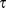 on 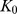 (Fig. 3A). When 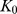 is small, the catalyst easily binds to substrates, whereas when 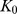 is large, the catalyst tends to be free. Hence, as 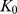 is increased, the threshold concentration of the catalyst at saturation is increased, and for larger values of 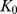, a sharp transition of 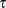 against the catalyst concentration disappears, as shown in Fig. 3A. Indeed, in the case of 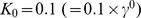, the relaxation process is gradually slowed by decreasing the catalyst concentration, and there is no sharp transition.

As 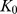 is decreased, the transition becomes sharper (see Fig. 3A for 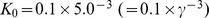 and 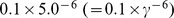). Indeed, for 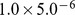 (Fig. 2), the transition from exponential to logarithmic relaxation occurs with a decrease in catalyst concentration below the critical concentration, 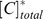. This critical value always remains at the total substrate concentration, as long as a sharp transition occurs. Below the critical concentration, 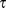 is proportional to 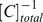, which is almost independent of 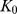.

#### (ii) The difference in the dissociation constants is important for slow logarithmic relaxation – condition for 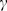


The relaxation behavior also crucially depends on the value of 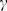 (Fig. 3B). For large 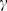, 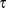 drastically changes at around the critical concentration of the catalyst. With an increase in 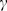, the slope of the logarithmic relaxation becomes smaller (Fig. S4), resulting in much slower relaxation. Above the critical concentration, 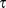 is independent of 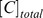 and below the critical concentration, 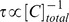. This transition is distinctively sharper than that of Michaelis-Menten type dynamics and is regarded as an example of ultrasensitivity [26].

In contrast, as 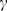 becomes smaller, the change in 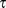 is reduced, and at 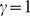, the logarithmic relaxation does not appear at all (see Fig. S2B and Fig. S4E) even at the low concentration of the catalyst. The relaxation time gradually decreases as the catalyst concentration increases and is almost constant if the catalyst concentration is higher than the substrate concentration.

#### (iii) With an increase in the number of modification sites, relaxation occurs more slowly – condition for 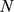


When the number of modification sites, 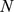, was increased, the gap in the relaxation time between the fast relaxation phase and the slow relaxation phase also increased (Fig. 3C). Indeed, in the fast exponential relaxation phase, the relaxation time was almost independent of 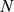, whereas in the slower logarithmic relaxation phase, it increased with 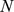. For 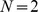, the relaxation process deviated from logarithmic relaxation, but occurred more slowly than in exponential relaxation (Fig. S4G). For 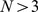, relaxation time increased exponentially with 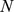.

### The order of relaxation reverses under the logarithmic relaxation regime

**Figure 4:**
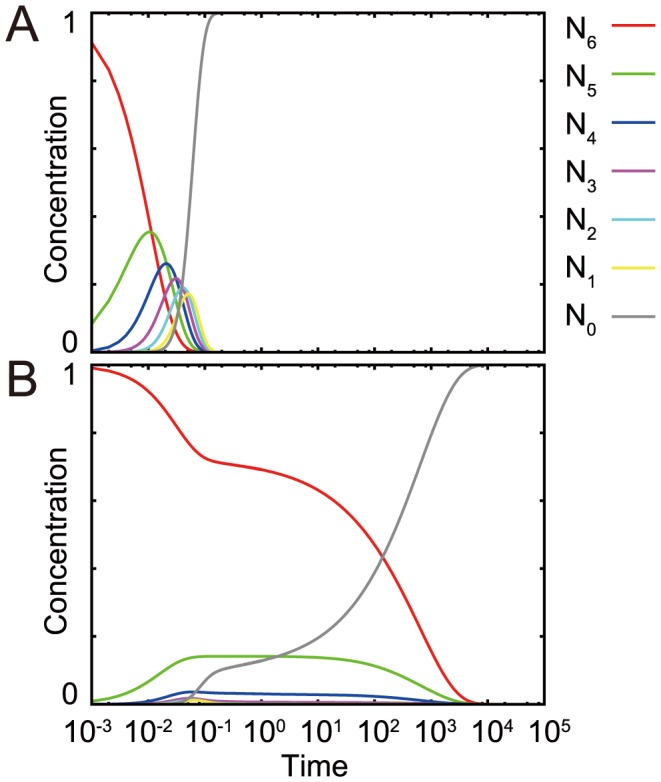
Relaxation process of each 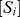. The time course of 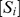 for each 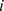 is plotted by setting the initial conditions as already described. (A) 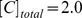 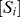
's relax in descending order, in the same manner as in the first-order reactions. (B) 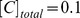 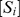
's relax in ascending order, that is, converse to the order expected from the first-order reactions. The highly modified state relaxes only after the relaxation of the less-modified 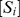. The relaxation process consists of several plateaus, which are typically observed in the relaxation process of kinetic glass [25].

The relaxation of the modification level is accompanied by changes in the modifications of substrate sites. Initially, 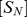 was set larger than other values for 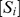, and the relaxation process to the stationary state with 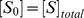 was investigated. At high catalyst concentrations, 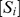 relaxed in descending order (Fig. 4A); decreases in 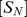 resulted in increases of 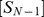, whose decrease resulted in an increase of 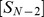 and so forth. This ordered relaxation agrees with that of first-order kinetics, which is expected given that a substrate with 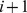 modified sites is demodified to a substrate with 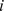 sites.

At low catalyst concentrations, however, the relaxation process is not ordered in such a monotonic manner. Indeed, highly modified substrates cannot relax readily (Fig. 4B), whereas less-modified substrates are able to take on modified states more easily. This relaxation of reversed order results from the limitation of catalysts and differences in catalyst/substrate affinity according to the number of modified sites 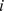, both of which underlie slow, logarithmic relaxation.

### The mechanism underlying logarithmic relaxation

Now we theoretically estimate the slow relaxation dynamics. Here, the relaxation dynamics, even after elimination of the variable 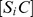 by assuming fast equilibration of binding and unbinding dynamics, are complicated and nonlinear to be solved analytically.

Note that slow relaxation requires competition for a catalyst, which is present at low abundance, and heterogeneity of affinity between substrates and the catalyst. Because of these two conditions, the time scales of relaxation for each modified substrate are separated, which slows the relaxation of modification dynamics. Here, we roughly estimate the long-term relaxation dynamics asymptotically, by approximating it by superposition of the eigenmode relaxation dynamics, which are approximated by relaxation of each modification level 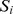. In our case, the affinities between substrates and a catalyst are distributed exponentially, and thus the time scales of demodifications, accordingly the eigenvalues, are also distributed exponentially. As for the estimate by superposition, we follow the scheme adopted in the theory for slow relaxation dynamics in glass (see e.g., [25]). Then, in the limit of large 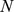 and 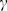, with small 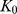, the total relaxation dynamics, given as a summation of such demodification dynamics of abundant substrates, are estimated to be logarithmic.

To estimate the relaxation dynamics, we first focus on the dynamics of substrates consisting of only a single demodification reaction, i.e., 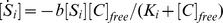. When 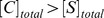 and 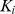 is sufficiently small, the abundance of free catalyst is larger than 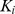; therefore, the dynamics of substrate abundance are estimated by
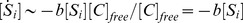
(3)


Thus, the relaxation of substrates is exponential and independent of 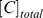. In contrast, when 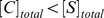, almost all catalysts are bound to substrates to form a complex form; therefore, that the abundance of free catalyst molecules approaches zero. Hence, 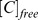 is smaller than 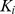. In this case, 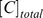 is estimated as 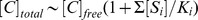. Here, in the large 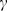 limit, 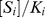 for 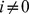 is negligible; thus, 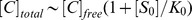. When 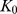 is sufficiently small, 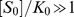; therefore, 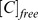 is estimated by
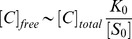
(4)


Thus, the dynamics of 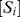 for 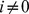 are given by
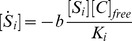


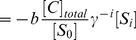
(5)


Here, the total modification level is given by 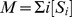.

Although the dynamics of the total modification level depend on the initial condition and both the influx and efflux of each 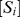, the eigenvalue of each 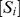
's relaxation dynamics is mainly governed by the efflux, because a contribution to the eigenvalue of the influx is given as 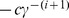 and of the efflux is given as 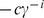, where 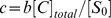. Hence, a contribute of the influx is negligible for large 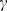, thus the eigenvalue of each 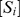 dynamics is governed by 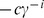. Then, the time evolution of the total modification level is given by 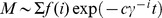, where 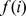 is the fraction of each eigenmode. Therefore, in the low catalyst concentration regime, the relaxation time depends on the inverse of 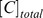 and is determined by 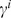 for maximal 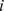; therefore, when the number of modification sites, 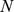, increases, the relaxation time increases in proportion to 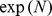. In the large 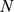 limit, the summation of 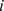 can be estimated by integration as 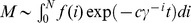. When there is no singular dependence of 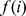 on 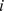 (i.e., the initial condition should not have singular dependence that is quite exceptional as in 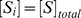 for 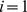 and 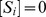 for 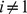), by setting 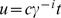, the integral is calculated as
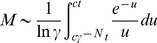


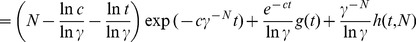
(6)


Here, the divergence of 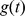 and 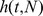 as 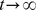 and 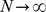 is considerably slower than the exponential. When 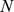 is sufficiently large, 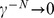 such that
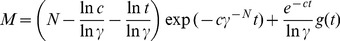
(7)


Therefore, the 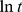 dependence is obtained asymptotically for large 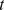 when 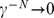. The above estimation suggests that a small 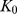 is needed for switching between fast and slow relaxation, and a large 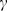 is needed for slower relaxation that allows separation of the time scale of each substrate, and a large 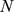 is needed for logarithmic relaxation. Thus, when 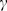 and 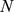 are large and 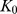 is small, the time evolution of protein modifications is expected to asymptotically follow a logarithmic pattern. Our simulation results show that the relaxation is much slower than exponential and does not follow the logarithmic form perfectly, because 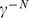 is small but finite (see Fig. S2B).

The slowing of relaxation caused by competition for the catalyst is also intuitively understood. When the 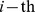 modified molecules are demodified, the amount of 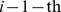 modified molecules will increase. Because the 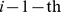 modified molecule has stronger affinity for the catalyst than the 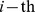 modified molecule, the binding of the 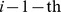 modified molecules to the catalyst hinders the binding of the 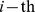 modified molecules. Thus, the demodification process is slowed depending on the modification level with an increase of the timescale as 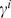 for the 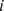-modified substrate. Therefore, the order of relaxation becomes reversed in the logarithmic regime (see Fig. 4B).

The above slowing mechanism resulting from the summation of distributed exponential relaxation dynamics is studied as the slowing of the equilibration process of glass. In particular, the mechanism we describe here is identical to that previously proposed for a chemical glass in catalytic networks [25], where a negative correlation between the abundance of a substrate and that of its catalyst suppresses the relaxation. In contrast to the abstract catalytic network model, our study adopts a realistic protein model with modification sites; therefore, input signals are easily administrated, storing information, and erasure of memory is achieved with applicability to the present cells.

### Continuous memory as dependence of the relaxation time or modification level on the input

**Figure 5:**
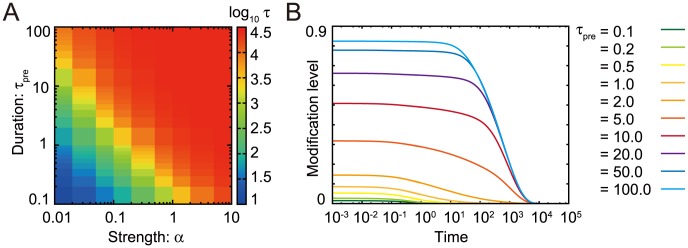
Dependence of the relaxation process 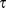 on the magnitude and duration of a stimulus. (A) The relaxation time after exposure to the stimulus with various magnitudes and durations is plotted as a color map. The initial condition is given as 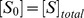 and 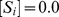 for 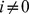, and the input is given as 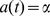 for 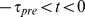 and 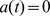 for 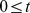. When the magnitude (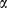) and duration of the stimulus (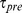) increase, 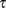 increases continuously over an order of magnitude. The catalyst concentration is set at 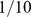 of the substrate concentration. (B) Dependence of the relaxation process on the duration of stimulus exposure. The duration of stimulus exposure is changed while the magnitude is fixed at 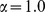. Here, the relaxation time increases nearly exponentially with the increase in duration for the some extent small 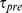. When 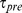 is sufficiently long, the modification is maintained for a long time.

When kinetic memory is formed via logarithmic relaxation, the relaxation time is not constant; rather it is instead further increased with the magnitude and the duration of stimuli given as 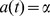 for 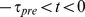 and 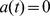 for 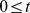, as shown in Fig. 5A. Moreover the maximal modification level also depends on the magnitude and duration of stimuli (Fig. 5B and Fig. S3). Thus, information regarding the input stimulus (i.e., magnitude and duration) is “memorized” as the difference between the relaxation time and the modification level. This continuous memory is in contrast to the on-off type of attractor memory.

Both the relaxation time and the modification level are candidate mediators of memory storage, as cells can access both of these mechanisms depending on the output pathway from the modification level. If cells use threshold dynamics as an output pathway and if the temporal integration of such output product contains information, then the relaxation time will be important for cells to decide their fate. In contrast, if cells use the modification level itself as an output pathway, the modification level will be more important. Depending on the output pathway, either the relaxation time or the modification level provides a candidate mechanism for useful information to be stored.

### The kinase-phosphatase model exhibits the same features as the chained modification model

**Figure 6:**
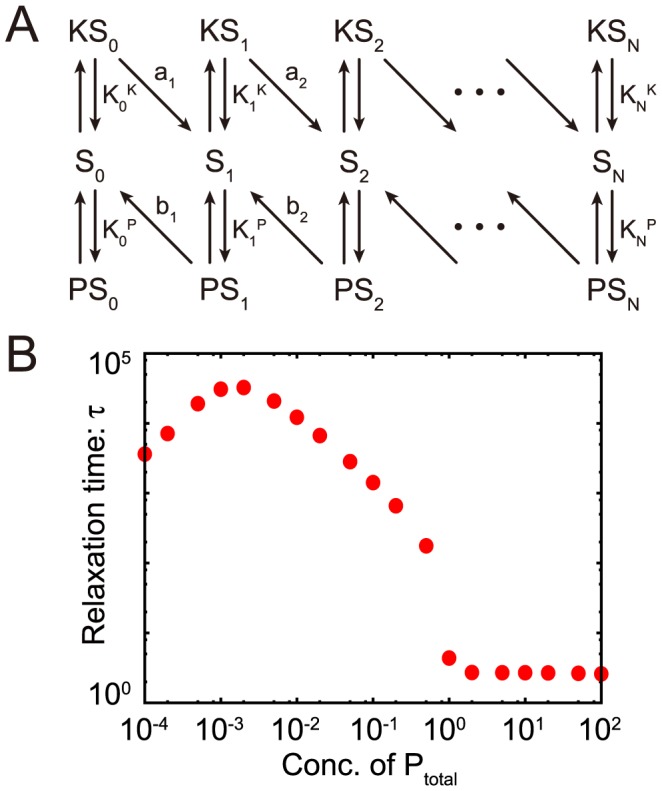
Kinase-phosphatase model (A) and its relaxation time (B). The relaxation times 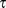 of the variables 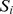 are plotted against the total concentration of phosphatase. 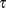 is defined as the time when the summation of 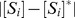 of all 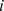 falls below the threshold value, after relaxation at a kinase-rich condition (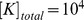). The model shows the transition from fast exponential relaxation to slow logarithmic relaxation at the critical point (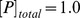). (When the amount of the phosphatase is lower than that of kinase (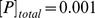), the relaxation time itself is shorter, whereas the logarithmic relaxation remains. Here, the stable fixed-point value of the concentration 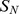 changes to a higher value, and the relaxation time is decreased.)

To further demonstrate the utility of kinetic memory, we also investigated the kinase-phosphatase (K-P) model (Fig. 6A). This model contains three components, i.e., kinase 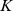, phosphatase 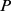, and substrate 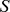, with multiple modification sites [27]–[29]. These modification states are characterized by 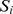, which denotes the number of phosphorylated residues 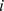. Kinases mediate an increase in the number of phosphorylated residues, whereas phosphatases facilitate inverse reactions. Generally, substrate modifications lead to changes in the affinities of substrates and catalysts. The dissociation constant between 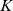 and 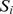 and that between 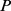 and 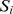 increase exponentially as 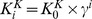 and 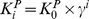, respectively, identical to the effect observed in the chained modification model.

We studied the relaxation process of 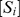 after the amount of the total kinase (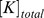) was varied, which functions as the input stimulus. We analyzed the relaxation process following the input of stimuli with a higher concentration of active kinases for a sufficient length of time. Here, again, the dephosphorylation processes in the K-P model show slow logarithmic relaxations when 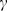 is positive. As shown in Fig. 6B, when the total amount of phosphatase is lower than that of the substrate, the dephosphorylation process shows very slow relaxation. The transition between fast and slow relaxation occurs at the point where the amount of phosphatase and that of the substrate is balanced.

### The extended Asakura-Honda model shows the same behavior as the chained modification model

**Figure 7:**
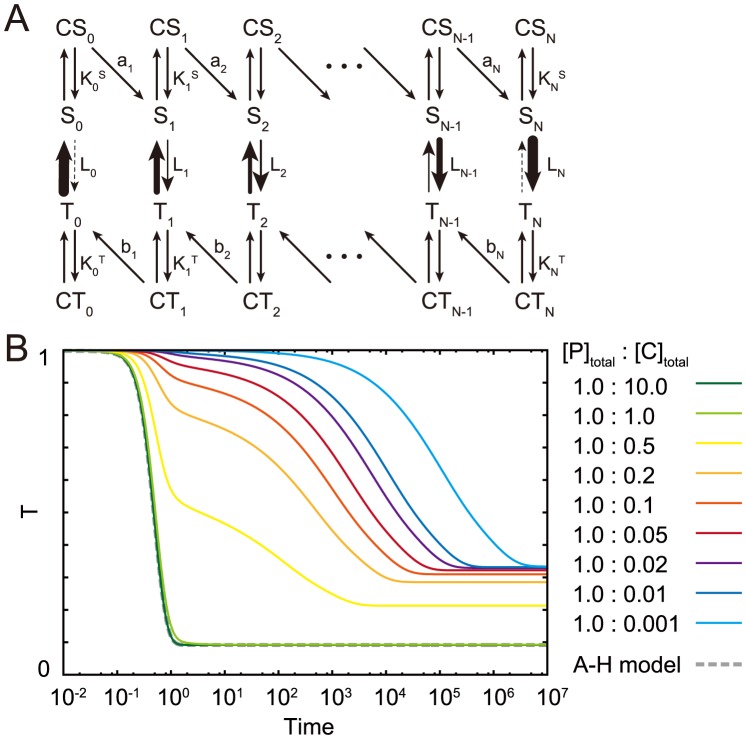
The extended Asakura-Honda model (A) and its slow logarithmic relaxation after exposure to an environmental stimulus (B). After the system is relaxed in the presence of the attractant as 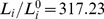, the system transitions to a repellant condition as 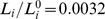, and the relaxation process of 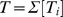 is computed. The parameters are given as 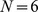, 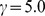, and 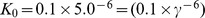. The time courses of 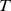 for different values of the catalyst concentration, 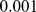, 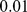, 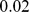, 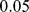, 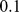, 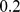, 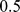, 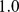, and 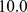, are plotted with different colors, where the concentration of 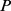 is fixed at 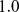. Although exponential relaxation is observed as in the original A-H model (dotted line), when the concentration of the catalyst is sufficiently large, the relaxation is drastically slowed as the concentration of the catalyst becomes lower than that of the substrate.

As another example, we studied an extended version of the Asakura-Honda (A-H) model. The original A-H model was introduced to explain processes of adaptation to changes in the concentration of external signal molecules (attractant and repellant) in chemotactic behavior [30]. This model represents a two-state receptor with multiple modification sites. Receptors in different states are recognized by distinct enzymes that facilitate an increase or decrease in the number of modified sites. In the model, the enzymes are always maintained at sufficient levels so as to not be rate-limiting. This A-H model consists only of first-order reactions, without Michaelis-Menten type reactions. Here, to discuss kinetic memory, we explicitly took the dynamics of the co-factor as a catalyst that catalyzes each modification reaction into account (Fig. 7A).

In the present paper, we introduced three models, i.e., a chained modification model having a single-state substrate and one catalyst, a kinase-phosphatase model having a single-state substrate and two catalysts, and a modified Asakura-Honda model having a two-state substrate and one catalyst. To demonstrate that the kinetic memory functions for all of these cases, we studied the A-H model here.

We analyzed the adaptation process after the input stimulus was applied to change the fraction of two states, and we found that in the extended A-H model, slow logarithmic relaxation occurs when the abundance of catalyst is limited, as observed in the chained modification model (Fig. 7B). The modified state memorizes the input amplitude and duration during the process of adaptation, and the conditions for this kinetic memory are essentially identical to those of the chained modification model (see Supporting Information and Fig. S5–S7). It is noted that a slow process exists only in the relaxation in the adaptation; the response remains fast independently of the parameters and the abundance of catalysts. The time scales for the response and relaxation are separated, and they are independently controlled by tuning the dissociation constants between the substrates and catalyst.

## Discussion

### Transition to long-term kinetic memory

In the present study, we evaluated three models, i.e., the chained modification model, the kinase-phosphatase model and the extended A-H model, which consist of a substrate with multi-modification sites and a catalyst that facilitates modification of the substrate's sites. As shown in Fig. 3 and Fig. 6B and Fig. S6, all of these models reveal a transition from fast exponential relaxation to slow logarithmic relaxation at the point where the concentration of the catalyst falls below that of the substrate.

The conditions for this transition are summarized as follows: There must be heterogeneity in the binding affinity of the catalysts that depends on the number of modified sites, such that the affinity for highly modified substrates should be sufficiently small. This leads to two requirements in our model in which the dissociation constant is set at 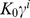 for the modification sites 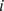.

(i) low 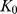 value: this supports a high affinity for substrates, such that the competition for catalysts among substrates is induced. To satisfy this requirement, 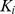 should be smaller than the substrate concentration 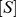 for all 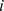; thus, the upper limit of 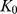 is restricted as 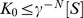.

(ii) 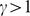: affinity depends on the number of modified sites on the substrates. When the above conditions are not satisfied, the relaxation time follows that of the Michaelis-Menten equation. In contrast, when 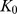 is small and 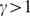, the relaxation changes drastically at the point where the concentrations of the substrate and the catalyst coincide. This change is much sharper than that expected from the Michaelis-Menten equation and is an example of ultrasensitivity [26].

When the concentration of the substrate (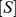) is lower than that of the catalyst (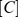), the concentration of a catalyst-substrate complex (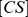) becomes approximately identical to that of the substrate (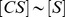), whereas when 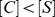, 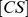 approaches 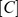. When the concentration of the catalyst decreases to levels below that of the substrate, various modified forms of the substrate compete for catalyst molecules. As a result, a transition to the slow relaxation occurs, induced by this enzyme-limited competition.

### Comparison between kinetic memory and attractor memory

The kinetic memory described here is expected to be advantageous over attractor memory with regards to the housekeeping cost. To maintain attractor memory, continuous invertible reactions are necessary, which consumes housekeeping energy [31]. Kinetic memory mediated by protein modifications also incurs housekeeping costs because of its irreversible modification reactions. However, during slow relaxation in kinetic memory, reversible association and dissociation reactions progress, but irreversible reactions are suppressed. Hence, the housekeeping costs in kinetic memory are expected to be lower than those for attractor memory.

Moreover, the memory erasure mechanism is different from that involved in multistable memory. The memory of a stable attractor is almost always constant except for the short time span needed for a switch to a different attractor. Erasure in kinetic memory, in contrast, is achieved by simply increasing the abundance of the enzyme, and thus could require a lower cost.

Attractor memory, however, may have some advantage with regards to the stability of the memorized state against noise and the constancy of the memorized state.

### Relevance of kinetic memory to cell biology

The kinetic memory we studied here is generated by a substrate with a few modification sites and a catalyst (enzyme) shared by each of the different modification states, resulting in enzyme-limited competition (ELC) [32], [33]. Hence, multimeric proteins, for example, can provide a molecular basis for kinetic memory. A candidate for a multimeric protein bearing kinetic memory is CaMKII, which forms a dodecameric structure with phosphorylation sites in each monomer [7]. It is known that phosphorylation of sites on CaMKII plays a critical role in the maintenance of early LTP. CaMKII is phosphorylated with increases in 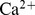 concentration and is dephosphorylated by protein phosphatase 1 (PP1). After the 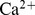 concentration decreases, phosphorylation levels remain high; therefore, CaMKII stores memory in its phosphorylation state. Indeed, if PP1 is limited, ELC among CaMKII molecules is expected to lead to kinetic memory, according to our argument presented here. In fact, it has been reported that the concentration of PP1 is lower than that of CaMKII in the postsynaptic density, as suggested by the condition for ELC [17]. As already described in the introduction, the phosphorylation state of CaMKII may not have multistability [18].

To confirm our “kinetic memory” hypothesis, it is important to analyze the time evolution of dephosphorylation of CaMKII over a long time course. If the dynamics of CaMKII dephosphorylation are slowed and distinguishable from a simple exponential, our hypothesis may be supported. Moreover, analysis of mutants that mimic phosphorylated and unphosphorylated states may also be effective. By using phosphorylated and unphosphorylated mutants, the difference in binding energy between phosphorylated and unphosphorylated states may be determined biochemically. Such results are helpful to determine the actual 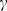 of CaMKII and how the relaxation dynamics depend on 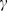.

Another candidate for kinetic memory may be CREB in brain synapses, which is known to mediate potentiation through altered phosphorylation levels. In late LTP, CREB is phosphorylated by CaM-dependent kinase and is gradually dephosphorylated by calcineurin, whereas phosphorylation of CREB leads to activation of gene expression [8], [34]. Here, it is reported that with increased duration of input, the relaxation time for dephosphorylation of CREB is prolonged, which is consistent with our kinetic memory [34].

It could also be expected that several other proteins with multi-modification sites may provide kinetic memory in our scheme. For example, the phosphorylation level of ERK, which has multiple phosphorylation sites, is elevated over a long time span after a transient increase in the level of nerve growth factor [9]. Such long-term phosphorylation may be a result of logarithmic relaxation in kinetic memory.

In a multistability (attractor) model, the memorized states are discrete and few in number, as the number of attractors typically increases only linearly with the number of modification sites. In contrast, kinetic memory can store continuous information regarding inputs, as we have discussed above. Indeed, cellular memory refers to the process by which organisms integrate information from continuous external conditions and retain it in their cells. Unicellular protozoa *P.caudatum*, when placed on a temperature gradient, accumulate in a region maintained at the previous cultivation temperature; this memory of the cultivation temperature is stored over approximately 40 min [3], [35]. Similar temperature memory in nematodes *C.elegans* over several hours has also been observed and is suggested to be stored at the level of a single thermosensory neuron [36]. It was also reported that *C.elegans* can memorize the NaCl concentration at the level of a single neuron [37], [38]. The kinetic memory scheme may shed light on such “continuous” cellular memory.

An important condition for kinetic memory is competition for the catalyst. The relevance of ELC to cellular functions has recently been discussed. For example, our previous study suggested the importance of ELC for temperature compensation of the period of biological clocks [32], [33]. Both in that study and in the present study, we found that distributed affinity leads to slow dynamics, and non-linear dependence of the reaction rate on substrate and catalyst concentrations is essential. Further applications of ELC to other cellular functions will be revealed in the near future.

Ultimately, it will be important to experimentally verify the kinetic memory hypothesis that we have proposed and tested here. Steps toward this aim should include measurements of relaxation processes for protein modifications under various catalyst concentrations. In addition, the development and use of mutant proteins that are able to mimic substrates with several modification states would enable the testing of varying affinities between modification sites. Such experiments would not only validate our model but also facilitate future investigations seeking to uncover mechanisms underlying primitive forms of cellular memory.

## Models

### The kinase-phosphatase model

The whole reactions are described below.
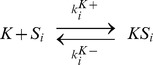


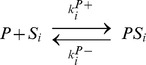


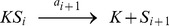


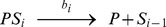



By assuming that association and dissociation reactions are faster than the other reactions, we obtained the following equations:
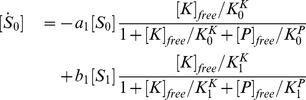


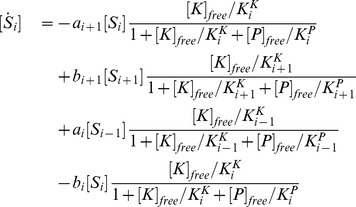


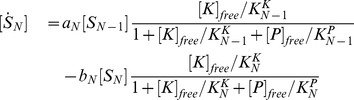
(8)


The total concentrations of the kinase and the phosphatase are conserved quantities, and thus
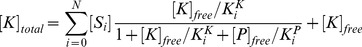
(9)

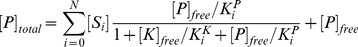
(10)


For parameter values, see Table 2 in Text S1.

### The extended Asakura-Honda model

The complete set of reactions in the extended Asakura-Honda model is given as follows:
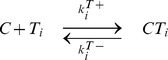


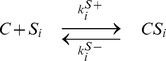


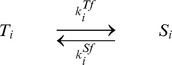


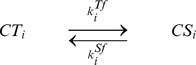


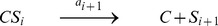


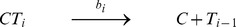



Assuming that catalyst association and dissociation reactions, in addition to flip-flop reactions between S and T, are much faster than modification reactions, they can be eliminated adiabatically; therefore, the model can be described as:
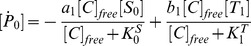


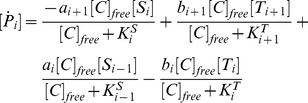


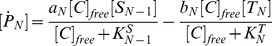
(11)


Where 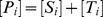 and 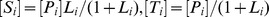. Here, 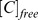 is the free catalyst that is not bound to 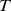 or 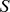, which satisfies
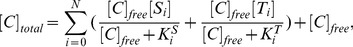
(12)where 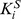 and 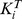 are dissociation constants between 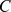 and 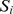 or 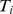, respectively. We assumed that the affinities between the catalyst and substrate decrease exponentially with the number of modified sites in the substrate, that is, the dissociation constants 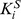 and 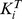 increase exponentially. Exponential increases of 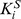 and 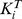 are required for perfect adaptation when the amount of the catalyst is sufficiently low (see Supporting information). In addition, we assumed that the dissociation constants are not different between the 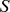 form and the 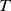 form; therefore, the dissociation constants are described as 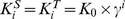. However, this is only for simplicity and is not essential. Stimuli are given as changes in 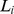, identical to the original A-H model.

For parameter values, see Table 3 in Text S1.

## Supporting Information

Figure S1 **Schematic representation of the bistable memory and kinetic memory.** (A) Parameter dependence of bistable fixed points and its representation by a potential landscape. The bistable memory is achieved as a double-well potential. Although the depth of each well corresponding to the stability of the state becomes shallower against the changes in the parameter, the modification remains at almost the same level. However, when the parameter exceeds the bifurcation point, one of the modification states loses stability and jumps to the other state. Thus, the bistable memory shows hysteresis against parameter changes. (B) Schematic representation of kinetic memory. Kinetic memory can be achieved as a single-minimum potential. When the catalyst concentration is low, the slope of the potential is very weak and the modification level changes slowly to the stable fixed point value. In contrast, when the catalyst concentration increases, the slope of the potential becomes steep and the modification level changes rapidly.
(EPS)

Figure S2
**Time evolution of the modification level.** The initial conditions and parameters are same as in Fig. 2. (A) The time courses of the reversed sign of the logarithmic modification level 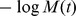 are plotted by using a log-log scale for different values of the catalyst concentration, 0.001, 0.01, 0.1, 1.0, and 10.0, with different colors, where the concentration of 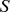 is fixed at 1.0. The gray dotted line indicates 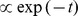. If the modification level follows 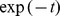, the plot is linear as in the gray dotted line. This is true when the concentration of the catalyst is higher than that of the substrate. However, when the concentration of the catalyst is lower than that of the substrate, the plot cannot be fitted by the linear form and changes more slowly than the exponential of the stretched exponential form. (B) The time courses of the modification level are plotted for different values of 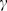, 10.0, 5.0, 3.0, 2.0, and 1.0, with different colors, where the concentration of 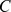 is fixed at 0.001. The abscissa denotes the time in a log-scale, and the ordinate shows the modification level. The gray line indicates 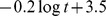 and the gray dotted line indicates 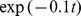. For 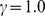, the relaxation curve is nearly exponential. For larger 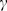, however, the relaxation curve cannot be fitted by exponential form (Note that in this plot by 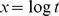, any exponential form 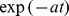 with any 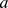 values is obtained just by adding a constant to 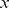, i.e., by shifting the horizontal axis), but approaches gradually the logarithmic form as 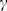 is increased.(EPS)

Figure S3
**Dependence of the relaxation process of the chained modification model on the magnitude of the stimulus.** The magnitude of the stimulus is changed while the duration is fixed as 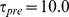. The increase in magnitude has a weaker effect on the relaxation time than the duration. The cofactor concentration is set at 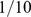 of the substrate concentration.(EPS)

Figure S4
**The relaxation process of the chained modification model for** 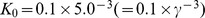 **(A),** 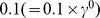 **(B),** 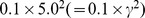 **(C) and** 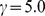 **,** 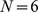 **,** 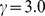 **(D),** 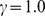 **(E) and** 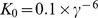 **,** 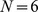 **,** 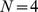 **(F),** 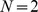 **(G) and** 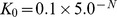 **,** 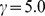 **.** The time course of the phosphorylation level is plotted. The initial condition is the same as in Fig. 2. Plotted for different values of 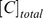 with different colors. The transition from fast exponential to slow logarithmic relaxation is sharp for low 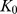. For a low 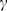, slow logarithmic relaxation becomes similar to fast exponential relaxation, and finally slow logarithmic relaxation disappears when 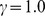. When the number of modification sites is low, the slow logarithmic relaxation becomes unclear.(EPS)

Figure S5
**Dependence of the relaxation process of the extended A-H model on the magnitude and duration of a stimulus.** (A) The relaxation time after exposure to stimuli with various magnitudes and durations is plotted as a color map. The initial condition is given as 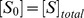 and 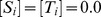 for other 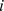. When the magnitude (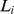) and duration of the stimulus increase, the relaxation time 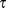 increases continuously over an order of magnitude. The catalyst concentration is set at 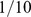 of the substrate concentration. (B) Dependence of the relaxation process on the duration of stimulus exposure. The duration of stimulus exposure is changed while the magnitude is fixed at 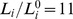. Here, the relaxation time almost increases exponentially with the increase in duration. (C) Dependence of the relaxation process on the magnitude of the stimulus. The magnitude of the stimulus is changed while the duration is fixed as 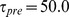. The increase of the magnitude has a weaker effect on the relaxation time than the duration.(EPS)

Figure S6
**Change in the catalyst dependence of the extended A-H model on the relaxation time** 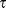 **against** 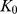 **, the dissociation constant (A), against** 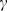 **, the heterogeneity of the dissociation constant (B), and against** 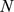 **, the number of modification sites (C).** 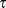 is plotted against 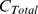; 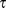 is defined as the time when summation of all of 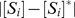 and 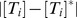 falls below the threshold value(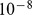) at the repellant condition (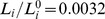), after a relaxation at the attractant condition (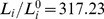). (A) 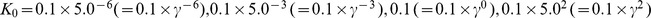, 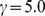, 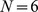. (B) 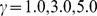, 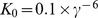, 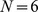. (C) 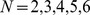, 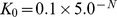, 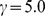.(EPS)

Figure S7
**Relaxation process for each** 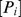 **of the extended A-H model.** The time course of 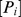 for each 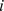 is plotted by setting the initial condition as already described. (A) 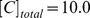 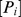
's relax in descending order, in the same manner as in the original A-H model. (B) 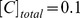 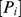
's relax in ascending order, that is, converse to that observed for the original A-H model. The highly modified state relaxes only after the relaxation of the lower modified 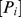. Relaxation of the highly modified 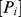
's plateau, a prominent feature of kinetic glass.(EPS)

Text S1 **Models and parameters.**
(PDF)
