# Supplementary material for: Kinetic Memory Based on the Enzyme-Limited Competition
Source: PLoS Comput Biol. 2014 Aug 14;10(8):e1003784. doi: 10.1371/journal.pcbi.1003784 (PMC4133053; doi:10.1371/journal.pcbi.1003784)
Supplement: Text S1 — Models and parameters. (PDF) [file pcbi.1003784.s008.pdf]

Text S1.

Kinetic memory based on enzyme-limited competition

Tetsuhiro S. Hatakeyama, Kunihiko Kaneko

1 Models and parameters

1.1 The chained modification model

In the simulation, we adopted the parameter values below unless otherwise noted.

a_i	a(t)
b_i	100.0 for all i
N	6
γ	5.0
K_0	$0.1 \times 5.0^{-6} (= 0.1 \times \gamma^{-N})$
$[S]_{total}$	1.0

Table 1: Parameter set of the chained modification model

We adopted the deterministic rate equation given by the mass-action kinetics, and it is simulated by using the fourth-order Runge-Kutta method.

1.2 The kinase-phosphatase model

In the simulation, we adopted the parameter values below.

N	2
a_i	1.0 for all i
b_i	10.0 for all i
γ	10.0
K_0^K	0.001
K_0^P	0.0001
$[S]_{total}$	1.0

Table 2: Parameter set of the kinase-phosphatase model

1.3 The extended Asakura-Honda model

The original Asakura-Honda (A-H) model was introduced to explain processes of adaptation to changes in the concentration of external signal molecules (attractant and repellant) during chemotactic behavior [1]. This model represents a two-state (S form and T form) receptor with multiple modification sites. The S and T form receptors are recognized by distinct enzymes, which catalyze an increase and a decrease in the number of modified sites, respectively. In the model, the enzymes are always maintained at sufficient levels so as to not be rate-limiting. $[S_i]$ and $[T_i]$ represent the concentration of the S and T forms of the receptor with i modified sites, respectively. When an external signal, such as a change in ligand concentration, is applied, $L_i = [S_i]/[T_i]$, the equilibrium

value of $[S_i]$ to $[T_i]$ is changed instantaneously. The equilibrium value of $[S_i]/[T_i]$ in the absence of an external signal is denoted as L_i^0, and that in the presence of signal is denoted as L_i. L_i increases upon administration of an attractant and decreases upon administration of a repellant. As the number of modified sites i is increased, the receptors are assumed to take on the T form more often, such that L_i^0 decreases with the number of modification sites i.

When the ligand concentration is changed, the total ratio $S/T = \Sigma[S_i]/\Sigma[T_i]$ changes initially. As the ratio increases (decreases), the number of modified sites increases (decreases). Subsequently, the highly modified receptors again flip to the T (S) form; thus, in both cases, the S/T ratio ultimately returns to the original proportion; therefore the A-H model exhibits perfect adaptation. By denoting the rate $S_{i-1} \to S_i$ as a_i and $T_i \to T_{i-1}$ as b_i, the fixed point concentrations $[T_i]^*$ and $[S_i]^*$ satisfy $-a_i[S_{i-1}]^* + b_i[T_i]^* = 0$ and $\mu_i \equiv [T_i]^*/[S_{i-1}]^* = a_i/b_i$ for all i. Under perfect adaptation $\mu_i = \mu$(const.) and $L_0^0 \gg 1$ and $L_N^0 \ll 1$: In this condition, $\mu = [T_1]^*/[S_0]^* = [T_2]^*/[T_1]^* = ... = [T_N]^*/[S_{N-1}]^* = (T^* - [T_0]^*)/(S^* - [S_N]^*)$ and $[T_0]^* \sim 0$, $[S_N]^* \sim 0$; therefore, $T^*/S^* \sim \mu$. At this point, perfect adaptation is achieved.

We adopt the A-H model for our study because it is a classic model for adaptation in which several modification sites can be considered. Indeed, various extensions to this model have been introduced to cover a wide range of adaptation phenomena [2, 3]. Here, for the purpose of modeling a slower relaxation than the original A-H model, we extend the A-H model to also explicitly include the concentration of a cofactor as a conservation quantity. Our extended A-H model has a cofactor that facilitates all reactions by acting as a catalyst and enabling the addition or removal of a functional (e.g., phosphate or methyl) group.

In the simulation, we adopted the parameter values below, in the case of $N = 6$. Following the original implementation of the A-H model, we initially set the system at an attractant condition (high L_i), in which the substrates were highly modified. We then exposed the system to a repellant condition (low L_i). Under these conditions, $T(= \Sigma[T_i])$, the fraction of methylated proteins increased transiently and relaxed to the original value.

a_i	1.0 for all i
b_i	10.0 for all i
L_0^0	$10^{5.5}$
L_1^0	$10^{1.5}$
L_2^0	$10^{1.0}$
L_3^0	$10^{0.5}$
L_4^0	$10^{0.0}$
L_5^0	$10^{-0.5}$
L_6^0	$10^{-4.5}$
$[P]_{total}$	1.0

Table 3: Parameter set of the extended Asakura-Honda model

It is important to note that in our extended model, the steady state value of T is different from that in the original A-H model, where the stable fixed point value of T is $T^* = \mu/(1+\mu) = a/(a+b)$. In the extended A-H model, however, μ_i is changed to $\mu'_i = \frac{a[C]_{free}/([C]_{free}+K^S_{i-1})}{b[C]_{free}/([C]_{free}+K^T_i)}$. Perfect adaptation requires that μ'_i is independent of i. This is possible in the limit of $[C]_{total} \to 0$ except in the case of $[C]_{total} \to \infty$ (original A-H model). In the case of $[C]_{total} \to 0$, $\mu'_i \sim \frac{a/K^S_{i-1}}{b/K^T_i}$. If the dissociation constants K^S_i and K^T_i increase with an exponent γi, μ'_i is independent of i as $\mu' = a\gamma/b$, and the extended A-H model shows perfect adaptation, with a change of the fixed-point value to $T^* = \mu_i/(1+\mu_i) = a\gamma/(a\gamma+b) = 1/3$. Generally, the steady-state concentration depends on the increase rate of the binding energy of the catalyst-substrate complexes, γ, except in the case of $\gamma = 1$, where the steady state value is constant, independently of the concentration of the catalyst.

References

1. Asakura S, & Honda H. (1984). Two-state model for bacterial chemoreceptor proteins. The role of multiple methylation. J. Mol. Biol *176*, 349–67.

2. Barkai N, & Leibler S. (1997). Robustness in simple biochemical networks. Nature *387*, 913–7.

3. Rao CV, Kirby JR, & Arkin AP. (2004). Design and diversity in bacterial chemotaxis: a comparative study in *Escherichia coli* and *Bacillus subtilis*. PLoS Biol. *2*, E49.
